# Attitudes of Older Adults in a Group-Based Exercise Program Toward a Blended Intervention; A Focus-Group Study

**DOI:** 10.3389/fpsyg.2016.01827

**Published:** 2016-11-22

**Authors:** Sumit Mehra, Tessa Dadema, Ben J. A. Kröse, Bart Visser, Raoul H. H. Engelbert, Jantine Van Den Helder, Peter J. M. Weijs

**Affiliations:** ^1^Digital Life Lab, Faculty of Digital Media and Creative Industries, Amsterdam University of Applied SciencesAmsterdam, Netherlands; ^2^Applied Psychology Lab, Faculty of Applied Social Sciences and Law, Amsterdam University of Applied SciencesAmsterdam, Netherlands; ^3^ACHIEVE – Centre for Applied Research, Faculty of Health, Amsterdam University of Applied SciencesAmsterdam, Netherlands; ^4^Department of Computer Science, University of AmsterdamAmsterdam, Netherlands; ^5^Department of Rehabilitation, Amsterdam Medical CenterAmsterdam, Netherlands; ^6^Centre of Applied Research, Faculty of Sports and Nutrition, Amsterdam University of Applied SciencesAmsterdam, Netherlands; ^7^Department of Nutrition and Dietetics, VU University Medical Center AmsterdamAmsterdam, Netherlands

**Keywords:** older adults, physical exercise, physical activity, daily functioning, technology, focus-group, mHealth, persuasive technology

## Abstract

Ageing is associated with a decline in daily functioning and mobility. A physically active life and physical exercise can minimize the decline of daily functioning and improve the physical-, psychological- and social functioning of older adults. Despite several advantages of group-based exercise programs, older adults participating in such interventions often do not meet the frequency, intensity or duration of exercises needed to gain health benefits. An exercise program that combines the advantages of group-based exercises led by an instructor with tailored home-based exercises can increase the effectiveness. Technology can assist in delivering a personalized program. The aim of the study was to determine the susceptibility of older adults currently participating in a nationwide group-based exercise program to such a blended exercise program. Eight focus-groups were held with adults of 55 years of age or older. Two researchers coded independently the remarks of the 30 participants that were included in the analysis according to the three key concepts of the Self Determination Theory: autonomy, competence, and relatedness. The results show that maintaining self-reliance and keeping in touch with others were the main motives to participate in the weekly group-based exercises. Participants recognized benefits of doing additional home-based exercises, but had concerns regarding guidance, safety, and motivation. Furthermore, some participants strongly rejected the idea to use technology to support them in doing exercises at home, but the majority was open to it. Insights are discussed how these findings can help design novel interventions that can increase the wellbeing of older adults and preserve an independent living.

## Introduction

The number of older adults in Europe will rise in the coming years (EUROSTAT, 2015). Ageing is associated with a decline in daily functioning and mobility (Walston et al., 2006; de Vries et al., 2012). A physically active life and physical exercise can minimize the decline of daily functioning (Nelson et al., 2007). Older adults with a sedentary lifestyle are at higher risk of health related problems, becoming dependent and facing a lower quality of live (King et al., 1998; Fried et al., 2001). Studies have shown that older adults benefit from regular exercise that increases muscle strength, balance, endurance, and flexibility (Taylor et al., 2004; Warburton et al., 2006). Physical exercise improves physical-, psychological- and social functioning and preserves an independent living (Taylor et al., 2004; Warburton et al., 2006; Nelson et al., 2007; de Vries et al., 2012).

To execute physical exercises correctly and persistently, however, knowledge, skills and tenacity is needed (Dishman, 1990). It has been argued that the Self-Determination Theory (SDT) is useful for understanding the initiation and maintenance of physical exercise behavior and is successful in predicting the adherence to exercise programs (Wilson et al., 2008; Teixeira et al., 2012; Silva et al., 2014). SDT poses that intrinsic or internalized extrinsic motivation is determined by the extent that three basic psychological needs are met (Ryan and Deci, 2000). Firstly, the need for autonomy. People need to feel in control of their behavior and goals they strive. Exercise programs should match the personal goals of an individual. Secondly, the need for competence. People need to gain mastery of relevant tasks and skills to achieve those goals. Guidance in executing exercises properly plays a key role. Thirdly, the need for relatedness. People need to feel connected to other people and have a sense of belonging. Exercising with peers can motivate to persevere. The beneficial role of supervision and peers is reflected by the higher adherence to supervised group-based programs then to programs where individuals are expected to exercise in solitude without supervision (King et al., 1998; Picorelli et al., 2014).

Although older adults can benefit from the supervision of an instructor and the company of peers, there are several disadvantages too. First of all, it is not always feasible. Employing an instructor and renting a location that can accommodate a group of participants can be costly. Secondly, in a group-based exercise program there is less opportunity to tailor to the individual needs of participants. Thirdly, participants need energy, time, and money to commute to the location. This is especially true for older adults that have, in general, more financial and physical limitations than others do. Because of those barriers the exercise frequency of supervised group-based programs is often too limited to attain health benefits (Dishman, 1990; Stiggelbout et al., 2004).

However, a combined intervention can compound the benefits of a supervised group-based exercise program with the benefits of an individual exercise program to achieve the required intensity, frequency and duration of exercises (King et al., 1998). The American College of Sports Medicine recommends exercising three to five times a week 30–60 min with moderate intensity (Chodzko-Zajko et al., 2009). Furthermore, technology, such as a computer, tablet, or smartphone can provide support for home-based exercises and tailor the program to the individual needs (Krebs et al., 2010; Webb et al., 2010; Brouwer et al., 2011).

To explore the attitudes of the older adults toward such a blended exercise program a qualitative study was carried out as part of the VITAMINE and MOTO-B project that intent to develop an intervention to increase the vitality and functional ability of older adults (>55 years of age) in the Netherlands. The aim of the study was to determine the susceptibility of older adults currently participating in a nationwide group-based exercise program to a personal tailored home-based exercise program supported by technology. The research questions were:

(a) What motives do older adults have to participate in a group-based exercise program?

(b) What are their attitudes and expectations toward doing additional exercises at home?

(c) What are their attitudes and expectations of supporting technology to facilitate home-based exercises?

To address the research questions focus-groups were held. In line with the SDT the results were analyzed in order to identify the motives, attitudes and expectations of older adults toward of blended exercise programs. The insights of this study will help the design of novel interventions that increase the health benefits of older adults and contribute to an independent living.

## Materials and Methods

### Design

Focus-group interviews are an effective means to understand what people feel or think about various issues (Krueger and Casey, 2014), including health related believes (Basch, 1987), and were therefore used in this study to infer the attitudes of older adults toward a blended exercise program. As prescribed by the methodology of focus-group interviews (Krueger and Casey, 2014), the recruitment of focus-group was ended when the saturation point was reached and no new information was presented.

### Participants

Participants were recruited by convenience sampling from a community-based program known as More Exercise for Seniors (in Dutch *Meer Bewegen voor Ouderen*, abbreviated as MBvO). In this nationwide program weekly 300,000 older adults of 55 years and older across the Netherlands participate in a weekly group-based exercise class under supervision of a certified trainer. An e-mail with information about the study and the goal of the focus-group was send to a number of trainers asking permission to pay a visit. During the visit the study was verbally explained to the older adults and were asked to sign-up for the focus-group that was to be held at a later date. Also flyers were given with additional information. Participants had to be 55 years or older and live independently in order to participate in the study.

The recruitment of focus-group was ended when saturation point was believed to be reached based on a preliminary analysis of the results. In total 15 trainers were contacted and eight focus-groups were held, including two pilots, with in total 48 older adults.

### Materials

An interview guide was developed according to the instruction manual for focus-group of the Dutch quality institute for healthcare (CBO, 2004). The interview guide was reviewed by a panel of experts with a diverse background, ranging from human movement to ICT. Two pilot focus-groups were conducted to test and refine the guide. The resulting interview guide is presented in **Table 1**.

**Table 1:** Interview guide.

**Introduction**
(1) What is your name and what is your mean reason to participate in MBvO?
(2) What do you think about MBvO?
**Transition**
(3) What are your activities beside MBvO?
(4) Which activities do you like?
(5) Which activities are the most important for you?
(6) What are the activities that are more difficult when you are older?
(7) What is the reason that activities are more difficult now?
(8) What motivates you to do activities and exercises?
(9) What are the barriers to do activities and exercises?
**Core**
(10) What is your opinion about an additional home-based exercise program?
(11) Do you want to do more activities or exercises? What do you like to do?
(12) Are you already doing exercises at home?
(13) What kind of exercises do you like to do?
(14) What do you think about exercising at home?
(15) Do you think you would do exercises at home?
(16) What are your requirements for a home-based exercise program?
(17) What motivates to participate in a home-based exercise program?
(18). What do you think you need to do exercises at home?
(19) What do you think about technology to support a home-based exercise program?
(20) Would technology help you to do activities and exercises?
(21) What are your requirements for technology?
(22) What do you think about different technologies?
(examples: video’s, music, virtual contact, wearables)
**End**
(23) What is your most important recommendation to develop an additional home-based exercise program?

### Procedure

The focus-groups took place at the locations where the weekly MBvO-class were held and at average consisted of six participants. During the focus-group a semi-structured interview and brainstorming session was held, guided by a moderator and an assistant that took notes. Prior to the focus-group the participants provided demographic information and signed an informed consent to agree to an audio-recording of the interview. The focus-group started with an introduction, informing participants that questions would be asked regarding their opinions about an additional home-based exercise program. It was emphasized that there were no right or wrong answers and that all opinions were equally valued by the moderator. During the focus-group the moderator asked questions, in line with the interview guide (see **Table 1**), and gave some examples when no responses were contributed. The focus-groups lasted approximately 1 h. The Medical Ethics Committee of the VU University Medical Center Amsterdam approved the study.

### Data Analysis

After each focus-group the moderator and assistant evaluated and discussed the most notable statements and common themes. The results of each focus-group were compared with other groups in order to find common patterns related to the research question. On basis of this preliminary analysis the researchers assessed if new information was obtained or saturation was reached. Afterward the assistant transcribed the audiotapes anonymously and the transcripts were analyzed with software for qualitative data analysis (MAXQDA). The transcripts were analyzed using a sequential coding strategy. Three types of coding are used consecutively: open, axial, and selective coding (Boeije, 2010). The initial codes (themes) were created by studying the segmented information and the assistant’s notes (open coding). Then the open codes were subcategorized to provide more details of each category and to indicate connections between different categories (axial coding). At the end the core categories were identified and matched with the three elements of the SDT; autonomy, relatedness, and competence (selective coding). All data was independently coded for themes by two researchers. When they differed in classifying participant’s remarks, a discussion was held in order to reach consensus. If they failed to do so, a third researcher decided which theme was most appropriate.

## Results

### Participants

Due to technical problems, the recording of one focus-group was lost and could not be analyzed. Furthermore, participants of the two pilot-groups were excluded from data-analysis. Data from the remaining 30 participants was included in the analysis and reported below.

The mean age was 74 years (*SD* = 9, range 58–88). The mean length of participation in the weekly MBvO-classes was nine years (*SD* = 9, range 3 months – 28 years). All participants were female (*N* = 30). The level of completed education the participants received varied, ranging from no schooling or elementary school (*N* = 3), lower vocational schooling, or lower secondary schooling (*N* = 12), intermediate vocational schooling or intermediate/higher secondary schooling (*N* = 13) to higher vocational schooling or university (*N* = 2).

### Motives do Older Adults to Participate in a Group-Based Exercise Program

#### Autonomy

Staying physically fit and being self-reliant was identified as a major reason for the participants to join the weekly MBvO classes. They indicated they considered themselves to lead an active lifestyle, doing household chores (washing windows, gardening, vacuuming, cooking, e.g.) and various leisure time activities (tennis, swimming, dancing, fitness, e.g.). Examples of typical remarks were: “…being on the move is for me reason number 1; I believe it is very important for staying healthy,” “…living independently is for me the main reason. It is crucial” or “…to be able to take a stroll on a nice day. To the forest or along the sea. Imagine that you can’t do that!”

Nevertheless, they believed doing additional exercises would contribute to their ability to remain mobile and live independently for a longer time. Some participants stated specific complaints as the reason for joining the exercise program (arthritis, e.g.). A few participants expressed clearly a fear of becoming dependent on others, as illustrated by the following remarks: “…the mere thought of being dependent on neighbors, friends, or whosoever. I wouldn’t want that,” “…I want to remain mobile, to buy groceries, cook and not have to rely on others,” and “…I am scared to death of becoming dependent on others. That is the worst thing that could happen to me.”

#### Competence

The majority of the participants stressed that they felt that expert guidance was crucial. The instructor that led the MBvO classes indicated the importance of each exercise and how it can be carried out safely. They clearly valued the supervision of the instructor, as illustrated by the following remarks: “I believe what plays a part is the guidance. Someone who tells you what you should do” or “…[the instructor]… how you should do it and what you are able and aren’t able to do.”

#### Relatedness

Besides staying physically fit, as mentioned before, social relations and ‘having fun’ were identified as other major reasons for the participants to join the weekly MBvO classes. Unanimously participants referred to this social aspect. They indicated they had good rapport with the group, felt that the presence of their peers motivated them to exercise and enjoyed each other’s company. In some cases participants even formed close friendships since they exercised across the years together. Typical remarks made by the participants were “for me it’s all about the social atmosphere” and “I wouldn’t want to miss out on being in touch with the others.”

### Attitudes and Expectations Toward Doing Additional Exercises at Home

#### Autonomy

Participants felt that a home-based exercise program could have several benefits. First of all, it could be tailored to their individual needs, allowing such a program to match their personal goals. Secondly, they indicated doing exercises at a moment that suits them, would also be a benefit compared to a group-based exercise program. Thirdly, participants were positive about the possibility of an exercise program that could be followed at different difficulty levels and was self-paced.

Typical remarks that illustrate the opportunities they identified were: “… a program with different difficulty levels, that allows you to take it each time further,” “…something like the television program The Netherlands on the Move (in Dutch *Nederland in Beweging*), but with the opportunity to adjust it to your pace” and “I belief having a choice is important and the pace, I should be able to decide for myself.”

#### Competence

Besides recognizing benefits of a home-based exercise program, the participants also expressed concerns if they would carry out exercises at home without guidance. Firstly, the felt the need for background information about the exercises. They indicated they would want to know which exercise contributes to which goals and if the exercise will improve strength, flexibility, balance, or endurance. They believed this information would motivate them to do the exercises. Secondly, the participants felt the need for instructions how to perform the exercises properly, thereby minimizing the chance of injuries. Some participants indicated that safety was an important concern. Thirdly, some participants mentioned that they need *structure*. To exercise at a specific day or time would help them to maintain their exercise routine. Fourthly, participants expressed the need for variation in a home-based exercise program as they valued this in the weekly MBvO classes. Fifthly, numerous participants indicated they considered themselves to have a busy life and felt that daily physical exercises should not take up more than 15 min a day.

Typical remarks that illustrate the competence related opinions: “I want to know which joints will benefit from a specific exercise,” “then it becomes enjoyable, because you know what the benefits are” and “I believe you should build up a routine, like it’s Monday so let’s get started!” and “I hava a lot of activities… such an exercise program should be 15 min at the most.”

#### Relatedness

The participants recognized the benefits of doing exercises at home in addition to the weekly group-based exercises, but expressed concerns if they would be able to motivate themselves to do so, as illustrated by the following remark: “…if I would get a list of exercises I should do at home, I would manage for 2 days, but that’s about it.” They felt the social support of the weekly classes were crucial. The participants expected to miss the peer pressure and the social control if they would do exercise at home. Some typical remarks were: “…I don’t think I would enjoy going about on my own” or “…I don’t believe that people that are active, will exercise at home without any guidance.” Some participants referred to the fact they had similar experiences with exercises that were prescribed by physiotherapists. Initially they would adhere to it, but in time their motivation drops and fail to maintain their home-based exercises.

A few participants raised, however, the idea of doing exercises with a spouse or with friends in order to mobilize social support in absence of a group led by an instructor, as illustrated by the remarks “… I might exercise with my husband” and “… if on Sundays I could go walking with a group instead.”

### Attitudes and Expectations of Supporting Technology to Facilitate Home-Based Exercises

#### Autonomy and Competence

When participants were asked if they believed technology like computers, tablets or smartphones could assist them in doing exercises at home, the opinions varied greatly. A few participants rejected the notion strongly, stating “…it doesn’t interest me,” “I think it’s awful” and “I oppose it. My children try to impose it, but I won’t have it!” Several participants indicated the fear of not being competent. They voiced a lack of confidence, even shame. Typical remarks were “…as an elderly, you don’t understand computers really and feel a bit dumb,” “… I don’t have any brains, so I never have learned it” and “…I feel embarrassed to ask my children for help.”

On the other hand, the majority of the participants were receptive to the idea of technology assisting them in home-based exercises and some were even enthusiastic. Typical remarks were “I have the feeling 1 day it’s bound to happen [using computers], I am the only family member who doesn’t have one,” “…I wouldn’t mind giving it a try,” “… I’m actually curious about it,” “I have never learned to type, but I have an iPad, that’s very easy” and “that would be fun!”

When the participants were asked how technology could support them in doing exercises at home, the responses ware limited. First thing that came to mind were videos of photo’s how exercise should be performed. Some participants, however, expressed concerns that exercises recommended by a computer or tablet would not know their physical limitations to the same extent as the instructor does, illustrated by the following remark: “…has been operated on her hip and then she says [the instructor], bending forward is ok, bending backward is ok, but don’t bend sideways…. She gives all those kind of instructions.”

#### Relatedness

If technology could not only support the participants in performing exercises, but also foster the social relationships they grew fond of in the weekly face-to-face classes, the participants held mixed opinions about. Some participants indicated they had no need for additional contact outside the weekly classes, while others stated they had already ample means if they wanted to reach out (“we can also pick-up the phone”). A few participants, however, came up with the notion to use video calls (Skype, FaceTime, e.g.) to form a virtual group and do exercises simultaneously at home. Finally, one participant welcomed video calls as a means to combat loneliness and stay in touch with others: “I have been thinking about it. I don’t only want to have enough exercise, but also to get by. You lose family, I don’t have kids, friends pass away, so you are left on your own. To be in touch with someone who lives on the other side of the county would be very nice…”

## Discussion

As outlined in the introduction, ageing is associated with a decline in mobility and daily functioning that is needed for independent living. Physical activity and physical exercise can prevent or limit the decline and improve the overall wellbeing of older adults. An intervention, in line with ACSM recommendations, that combines group-based exercises led by an instructor with home-based exercises will help to achieve the needed intensity, frequency and duration of exercises in a functional context. The aim of this study was to determine the susceptibility older adults currently participating in a nationwide group-based exercise program to such a blended exercise program. The results of this study bear insights which should be taken into account when designing novel exercise programs.

Firstly, the motives of older adults to participate in an exercise program seem to be twofold. Older adults strive to remain self-reliant. Exercises that contribute to their autonomy can count on their support, on condition that they receive proper guidance in which the benefits of each exercise is stressed and instructions are provided how exercises are to be performed correctly. Self-efficacy, outcome expectation and perceived benefits play a key role in the adherence to exercise programs (van Stralen et al., 2009). On the other hand forming social relationships amongst peers and being in touch with others seems to be another clear motive of older adults to join an exercise program. The need for relatedness should be taken into account when designing interventions promoting physical activity or physical exercise.

Secondly, older adults recognize a number of benefits of additional home-based exercises. It allows them to a greater extent to tailor the exercises to their personal goals and needs in the level of difficulty, speed and timing, compared to group-based exercises. However, as also noted by Picorelli et al. (2014), older adults have concerns missing the guidance they receive in instructor led group-based exercises. A home-based exercise program should have detailed instructions how exercises are to be performed correctly and safely. Furthermore, due to a perceived lack of time, also noted by others (Lees et al., 2005; Costello et al., 2011), the exercise program should be concise.

Finally, the role technology could play in supporting older adults to perform exercises at home is indistinct. Some older adults oppose the notion of using supporting technology for an exercise program in whatsoever form. However, this does not apply to all older adults. The majority does seem to be either enthusiastic or open to it, albeit they have no clear view or expectations about it.

The insights of this study should be interpreted with caution, however. Firstly, the choice to explore motives, attitudes and expectations with focus-groups relies on introspection and self-report. This implies that the participants in this study were able to consciously reflect on those aspects and accurately report them. As with all introspection techniques, this may not have been the case (Nisbett and Wilson, 1977; Wilson and Dunn, 2004). The authors believe this played especially a role with questions regarding the use of technology. Most of the participants did not seem to have any experience with ICT. The answers they gave were largely based on general views they held about it. Although this should not be disregarded, future research could extend the findings by presenting older adults detailed scenario’s with use cases or working prototypes in order to further investigate this matter.

Secondly, the focus-groups were led by either a psychologist with an ICT background or a human movement scientist. It was believed that the broad academic background of these researchers reflected the diverse key issues that play a role. In moderating the focus-groups the differences in academic background may, however, also have led to minor differences in which topics were elaborated upon. Yet the impact of those differences were minimized by the fact that the results of each focus-group were reviewed and analyzed by both researchers, safeguarding the comparability.

Finally, one should be careful in generalizing the results of this study to older adults by and large. In line with the aim of the study, attitudes toward a blended exercise program were explored amongst older adults currently participating in a nationwide group-based exercise program. It is reasonable to assume that these attitudes may differ from older adults that are not engaging in such activities. Furthermore, all the participants in this study were women and varied strongly in age and schooling. Although these characteristics of the sample are indicative of the targeted population of this study in which women are in the vast majority, is does raise questions how gender, age and education level influences the attitudes toward exercise and supporting technology amongst other populations of older adults. Reviews indicate that, in general, education has a positive correlation and age a negative correlation with exercise behavior (Rhodes et al., 1999; Trost et al., 2002). Those reviews also conclude that the majority of studies show that men are physically more active than women. In contrast, Mesters et al. (2014) reported in a recent study that for older adults in the Netherlands the opposite appears to be true; older women attain more physical activity than older men. The authors stipulate this may be the result of the type of activities Dutch men prefer, but cannot keep up at late age (i.e., outdoor sports). Women might also be more susceptible to the social support provided by the group-based program. Concerning differential attitudes toward supporting technology, a review by Or and Karsh (2009) shows that the majority of studies found a positive relation between education and acceptance of health information technology, but no effect of gender. The effects of age were found to be inconclusive. Some studies report a negative relation, while other studies report a positive relation or no relation at all between age and the acceptance of supporting health technology (Or and Karsh, 2009). Future research could expand upon the findings of the present study and clarify remaining issues by investigating different populations. By taking the attitudes of specific older adult populations into account, novel interventions can be designed that contribute to a healthy and independent living.

## Author Contributions

BK, BV, RE, and PW played a role in the conception of the study. SM and TD conducted the focusgroups (gathered data). SM, TD, and JV analysed and interpreted the data. TD, JV, BK, BV, RE, and PW revised the article. All authors gave final approvement to the manuscript and agree to be accountable for all the work.

## Conflict of Interest Statement

The authors declare that the research was conducted in the absence of any commercial or financial relationships that could be construed as a potential conflict of interest.
